# Reliability of a Sleep Quality Questionnaire for Use in Epidemiologic Studies

**DOI:** 10.2188/jea.JE20110107

**Published:** 2012-05-05

**Authors:** Jennifer Girschik, Jane Heyworth, Lin Fritschi

**Affiliations:** 1 Western Australian Institute for Medical Research, The University of Western Australia, Nedlands WA, Australia; 2 School of Population Health, The University of Western Australia, Crawley WA, Australia

**Keywords:** sleep quality, sleep duration, questionnaire, reliability, test-retest, epidemiology

## Abstract

**Background:**

The longer-term health impacts of poor sleep quality are of increasing interest, as evidence suggests that there are rising levels of sleep disturbance in the community. Studies have reported links between sleep quality and increased morbidity and mortality. However, the results of these studies are constrained by limitations in the measurement of sleep quality in epidemiologic studies. The Breast Cancer Environment and Employment Study (BCEES) has developed a sleep questionnaire that attempts to address some of the limitations of previous sleep questionnaires. The present study assessed the test-retest reliability of the sleep questionnaire used in the Breast Cancer Environment and Employment Study (BCEES).

**Methods:**

Subjects for this reliability study were women who were participating as controls in the BCEES study. Test-retest reliability was evaluated for individual items, using weighted kappa for categorical variables and intraclass correlation coefficients (ICC) and limits of agreement for continuous variables.

**Results:**

Most sleep questions showed good agreement, ranging from 0.78 to 0.45. The ICC was 0.45 (95% CI 0.32–0.59) for lifetime sleep loss per year and 0.60 (95% CI 0.49–0.71) for symptom severity.

**Conclusions:**

The test-retest reliability of the general sleep questions was good, and future epidemiologic studies of sleep could reliably expand the number of assessed domains of sleep quality. However, reliability decreased as increasing detail was required from participants about specific periods of sleep disturbance, and changes to the questionnaire are warranted.

## INTRODUCTION

Sleep is a necessary feature of human health. Although the short-term effects of poor sleep quality, such as tiredness, fatigue, loss of concentration, and injuries have long been recognized, the longer-term impacts have not been extensively studied. With evidence to suggest increasing levels of sleep disturbance in the community,^1^^–^^3^ these longer-term health impacts of poor sleep quality are of increasing interest. A small number of epidemiologic studies have investigated sleep quality and a range of long-term health outcomes, including obesity, diabetes, and cancer, and some have reported links between sleep quality and increased morbidity and mortality.^4^^–^^10^ However, the results of these studies are constrained by limitations in the measurement of sleep quality.

Sleep quality is a complex mix of attributes (otherwise known as domains), including quantitative aspects such as the duration of sleep, time taken to get to sleep (sleep latency), and times woken during sleep (arousals), as well as more subjective aspects such as depth, restfulness, and refreshment.^11^^,^^12^ The number of domains of sleep quality measured in sleep research is influenced by the type and scope of the study being conducted. As a rule, instruments that collect information on larger numbers of domains are limited to assessing short-term and/or recent exposures, which may be insufficient for epidemiologic studies investigating long-term health effects.^13^

To collect information on long-term exposures, epidemiologic studies have thus far limited the assessment of sleep quality to 1 or 2 questions. Most commonly, these studies ask about usual sleep duration,^14^^–^^17^ but some studies have asked about subjective quality,^7^^,^^8^ ease of getting to sleep,^6^^,^^9^^,^^10^ and use of sleep medications.^10^^,^^18^ While these questions have advantages for the data collection process, they limit the scope for exploring the true relationship between sleep quality and health outcomes.

The Breast Cancer Environment and Employment Study (BCEES) has developed a sleep questionnaire that attempts to address some of the limitations of previous sleep questionnaires by asking questions on a larger number of domains of sleep quality as compared with previous studies (including usual sleep duration on work and non-work days and subjective sleep quality) and by asking participants to identify and describe (frequency, duration, and symptoms) any periods of sleep disturbance persisting longer than 2 weeks over their lifetime.

Given the potential of sleep quality to impact on a range of health outcomes, and the complexities of measuring sleep quality, expanding the scope of sleep quality measurement is an important aim for epidemiology research. However, an expanded measure of sleep quality will only benefit research if it is also shown to be reliable. Thus, the aim of this study was to assess the test-retest reliability of the BCEES sleep questionnaire.

## METHODS

### Study population

Subjects for this reliability study were women who were participating as controls in the BCEES study, which was a 3-year population-based case-control study conducted in Western Australia (WA) that investigated the role of environmental and occupational risk factors in the development of breast cancer. Eligible control subjects were women aged 18 to 80 years living in WA between May 2009 and August 2011, and were identified through the electoral roll. Voter enrollment is compulsory in Australia, and the electoral roll is considered a good population sampling frame. BCEES control subjects had no prior diagnosis of breast cancer and were frequency age-matched to cases in 5-year age groups.

### Study procedures

Consenting BCEES participants completed the BCEES questionnaire, which contained demographic and sleep questions. Approximately 2 weeks after the BCEES questionnaire was returned, BCEES control participants were invited to take part in the sleep reliability study. The invitation included an information sheet, consent form, and questionnaire. Participants were asked to sign the consent form, complete the reliability questionnaire, and return it in the reply-paid envelope provided. Participants who did not return the consent form or questionnaire were considered to be non-consenters. This study was approved by the WA Department of Health Human Research Ethics Committee.

### Reliability questionnaire

The reliability questionnaire consisted of those sleep questions from the original questionnaire that were most relevant to the main study hypothesis. In addition, the questionnaire was slightly restructured to prioritize the most important questions and allow for clear layout and ease of completion. All but 1 question had categorical answer categories. No demographic information was collected on the reliability questionnaire.

The questionnaire included 8 sleep questions (including sub-questions) that asked about: previous diagnosis of sleep disorders, usual sleep habits (eg, falling asleep with the light on, wearing a mask while sleeping), and domains of usual sleep quality, including sleep duration on work and non-work days and subjective sleep quality (Table 1). In addition, the questionnaire included a table in which participants were asked to record information on up to 7 periods of sleep disturbance during their lifetime (including the age at which it occurred, how many years it lasted, and categorical answers regarding how often they experienced particular symptoms of poor quality sleep).

**Table 1.** Overview of the sleep questions included in the BCEES study and the frequency of responses from questionnaire 1

Question number	Question	Answer Categories	Frequency
1	Ignoring the last year, in your adult years, how many hours of sleep on **work** days did you usually get?	Less than 5 hours Between 5–6 hours Between 6–7 hours Between 7–8 hours Between 8–9 hours More than 9 hours	2 25 66 78 17 4

2	Ignoring the last year, in your adult years, how many hours of sleep on **non-work** days did you usually get?	Less than 5 hours Between 5–6 hours Between 6–7 hours Between 7–8 hours Between 8–9 hours More than 9 hours	3 15 51 75 41 7

3	Have you ever been told by a doctor or other health professional that you have a sleep disorder, for example insomnia, obstructive sleep apnea, restless legs or narcolepsy?	Yes No	18 176

4	Have you ever taken melatonin to help you sleep?	Yes No	12 180

5	Ignoring the last year, do you generally consider yourself to be a good sleeper? That is, do you fall asleep easily and sleep soundly?	Very good sleeper Fairly good sleeper Fairly bad sleeper Very bad sleeper	43 102 34 8

6	Ignoring the last year, did you ever fall asleep with the light on?	Never or almost never Sometimes Often Always or almost always	100 71 14 2

​ 6.1	​ - On those nights when you fell asleep with the light on, did you ​ usually leave it on all night?	Yes No	4 62

7	Ignoring the last year, did you ever sleep with a mask that covers your eyes?	Never or almost never Sometimes Often Always or almost always	174 9 3 0

### Statistical analysis

Test-retest reliability was evaluated for individual items, using weighted kappa for categorical variables.^19^

For the sleep table, 2 variables were calculated for each participant (based on information provided in the sleep table): lifetime sleep loss per year and symptom severity. Although a measure of lifetime sleep loss per year has not been previously calculated, the value of a cumulative sleep variable has been proposed,^20^ and the concept of cumulative exposure-times has been used in other areas of epidemiologic investigation, for example, pack-years and fiber-years for assessing cumulative exposure to tobacco smoke and asbestos fibers, respectively.

Lifetime sleep loss per year was calculated as a measure of the number of hours of sleep loss a participant had experienced per person-year. The hours of sleep loss for each period of reported sleep disturbance was calculated as the usual duration of sleep on work days minus the hours of sleep reported during the period of disturbance. We used the midpoint of each category because the usual hours of sleep on work days was a categorical variable. For the open-ended categories, we used 4.5 and 9.5 for the lowest and highest categories, respectively. Hours of sleep loss was multiplied by the number of days the sleep disturbance lasted and summed over each period of sleep disturbance for each individual. This sum was then divided by the participant’s age (as reported in the first questionnaire). For example, if a 50-year-old participant who usually slept 8 hours per night on work days experienced 1 period of sleep disturbance that lasted 2 years during which she reported getting 6 hours per night, she would have a lifetime sleep loss of 29.2 hours per year. To put sleep-loss years into context, a person sleeping 8 hours a night accumulates 2922 hours of sleep per year. A small number of participants reported a longer than normal duration of sleep during their period of sleep disturbance (particularly in association with depression). This created a negative value for their sleep hours, which ultimately reduced the value of their sleep loss per year. However, because the aim of this study was to assess reliability, rather than association, these values were included.

Symptom severity was a measure of the severity of symptoms experienced during periods of sleep disturbance. For each period of sleep disturbance, participants were asked to report the frequency (never or almost never, sometimes, often, always or almost always) of 4 symptoms of sleep disturbance (trouble falling asleep, waking up during the night, trouble getting back to sleep if woken, and waking up too early in the morning). Frequency was coded as 0 to 3 (never or almost never to always or almost always). The frequency of each symptom was summed within, and then across, each period of disturbance to create a single variable for each participant that ranged from 0 to 84. A score of 0 symptom severity corresponded to participants who did not experience any symptom for any period of sleep disturbance, and a score of 84 corresponded to participants who reported always experiencing all symptoms for the maximum number of periods (7) of sleep disturbance.

To test for mean differences in lifetime sleep loss per year and symptom-severity between the 2 time points, we used the Wilcoxon signed-rank test for matched pairs. To test the level of repeatability we used the 1-way intraclass correlation, or ICC (1, 1), and the limits of agreement (repeatability coefficient), or LOA(r), method.^19^^,^^21^^,^^22^ The repeatability coefficient differs from the standard limits of agreement method in that it uses the within-subject standard deviation from a 1-way analysis of variance to calculate the limit (rather than the standard deviation of the difference “between” the 2 time points).^21^ The limits represent the range within which we expect 95% of the differences between the 2 measurements by the same method to lie. The width of the LOA(r) should be judged according to clinical significance.^21^ The level of bias (average agreement) was calculated as the mean of the differences between time 1 and time 2. The dependence of the difference on the magnitude of the estimates was assessed by linear regression. All analyses were performed using Stata version 11.1 (StataCorp, College Station, TX, USA).

## RESULTS

Data collection started on 14 January 2011 and finished on 15 June 2011. During this time, 231 reliability questionnaires were sent and 195 were returned, a response fraction of 84%.

Table 2 shows the demographic features of the study population. The mean age was 60 years (range 30–79 years). Most participants (67%) were born in Australia or New Zealand, and 40% had completed high school.

**Table 2.** Characteristics of participants in the BCEES sleep reliability study (*n* = 195)

Characteristics	*n*	%
Age (mean, SD)	(60, 10.2)	
Country of birth		
Australia/New Zealand	130	67
United Kingdom/Ireland	42	21
Europe	9	5
Asia	8	4
Other	6	3
High school education		
<year 9 or equivalent	32	16
Year 10 or equivalent	62	32
Year 11 or equivalent	19	10
Year 12 or equivalent	78	40
Don’t know	4	2

Up to 9 people had missing data for the sleep questions. These people were excluded from the weighted kappa analysis for these questions (Table 3). Most sleep questions showed good agreement, and the strongest agreement (0.78) occurred for the sub-question that asked participants who reported falling asleep with the light on whether they left it on all night. The questions that asked about duration of sleep on work days and duration of sleep on non-work days showed the same agreement (0.71). Fair to moderate agreement was found for the questions that asked participants whether they had ever experienced a period of sleep disturbance in their life (0.60) and how many periods of sleep disturbance they had experienced (0.59). The poorest agreement was found for recall of whether participants had ever slept with an eye mask (0.45).

**Table 3.** Kappa scores for test-retest reliability of sleep questions in the Breast Cancer Environment and Employment Study sleep questionnaire

Usual sleep habits question (*n*)	Kappa	*P*-value
Duration of sleep on work days (*n* = 192)	0.71	<0.001
Duration of sleep on non-work days (*n* = 192)	0.71	<0.001
Ever been diagnosed with a sleep disorder (*n* = 194)	0.69	<0.001
Ever taken melatonin to help you sleep (*n* = 192)	0.62	<0.001
Do you consider yourself a good sleeper (*n* = 187)	0.74	<0.001
Did you ever fall asleep with the light on (*n* = 187)	0.64	<0.001
- Did you usually leave it on all night (*n* = 66)	0.78	<0.001
Did you ever sleep with an eye mask (*n* = 186)	0.45	<0.001
Ever experienced periods of sleep disturbance ​ in lifetime (*n* = 186)	0.60	<0.001
Number of periods of sleep disturbance ​ in lifetime (*n* = 184)	0.59	<0.001

Forty-three participants reported never having experienced a period of disturbed sleep in their lifetime, and 24 participants had missing data such that sleep loss per year and symptom severity variables could not be calculated. In addition, for the sleep loss per year variable, a review of the data revealed an outlier—with a difference in sleep loss per year of almost twice that of the nearest value—which was excluded from the analysis. Ultimately, 127 participants with a value for sleep loss per year and 128 participants with a symptom severity score who reported at least 1 period of sleep disturbance at either time point were included in the ICC and LOA analysis.

**Figure 1.**
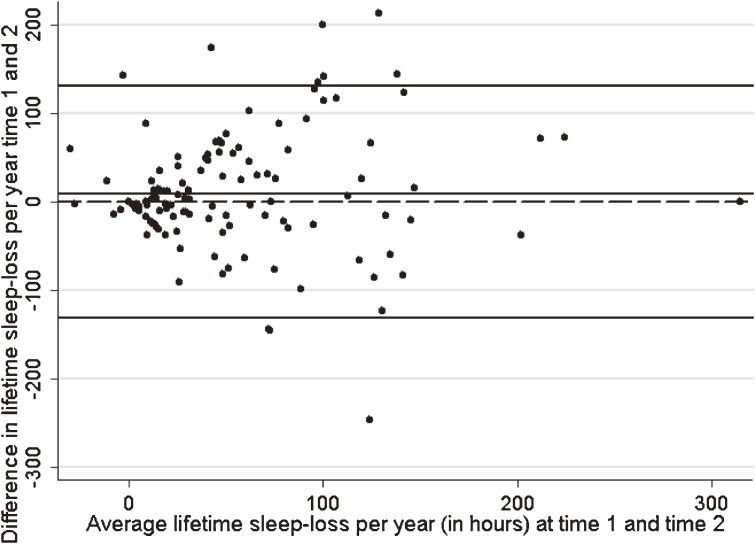
Limits-of-agreement (repeatability coefficient) plot of difference, by average sleep loss per year showing 95% LOA(r)

The Wilcoxon test showed no systematic difference between mean sleep loss per year at the 2 time points (*P* = 0.42). This was supported by the LOA(r) plot (Figure 1), which showed that the mean difference (bias) between reported sleep loss per year at time 1 and time 2 was not statistically significant (mean difference 8.74; 95% CI −2.96, 20.45). In addition, there was no evidence of dependence of the average bias between time 1 and time 2 as the amount of sleep loss per year increased: the β coefficient for the regression of the difference on the average sleep loss per year was 0.11 (95% CI −0.10, 0.33). The 95% LOA(r) ranged from −131.14 to 131.14, which indicates that even though, on average, the 2 estimates are the same, the sleep loss per year reported by an individual could vary from 131 fewer hours of sleep loss to 131 more hours of sleep loss per year from time 1 to time 2. To put this range into context, for a person who normally slept 8 hours a night (2922 hours of sleep per year), their reported sleep per year could vary from 2791 hours per year to 3053 hours per year). The ICC for lifetime sleep loss per year was 0.45 (95% CI 0.32, 0.59), which does not indicate strong agreement. Although the bias is small, and the dependence is not significant, the wide LOA(r) and the low ICC indicate modest agreement for the sleep loss per year variable.

**Figure 2.**
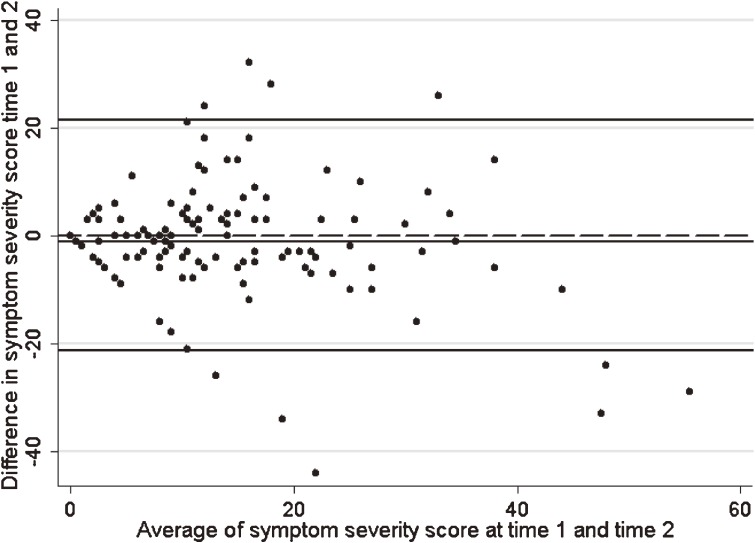
Limits-of-agreement (repeatability coefficient) plot of difference on average symptom severity score showing 95% LOA(r)

For symptom severity, the median score at time 1 was 11, with a range from 0 to 46 (out of a possible 84 points). The Wilcoxon test showed no systematic difference between mean symptom severity at the 2 time points (*P* = 0.16). This is supported by the LOA(r) plot (Figure 2), which indicated a small but nonsignificant level of bias between reported symptom severity at time 1 and time 2 (mean difference = −1.01; 95% CI −2.92, 0.90). That is to say, on average, people reported 1 less symptom at time 2 than at time 1. In addition, the ICC was 0.60 (95% CI 0.49, 0.71), which indicates moderate agreement. However, there was a very small dependence between the average bias between time 1 and time 2 as the severity score increased. The β coefficient for the regression of the difference on the average sleep loss per year was −0.19 (95% CI −0.36, −0.17), indicating that as severity score increased, the number of symptoms reported decreased slightly between time 1 and time 2. The 95% LOA(r) was wide, at −21.4 to 21.4, so although average symptom-severity scores were the same, the severity reported by individuals could vary from 21 points lower to 21 points higher from time 1 to time 2. Although the ICC was moderate and the bias was small, the significant dependence and wide LOA(r) indicate less than ideal agreement.

## DISCUSSION

Overall, the test-retest reliability of the sleep questions was good; however, it decreased as questions asked participants for increasing detail on specific periods of sleep disturbance.

Test-retest reliability of sleep questions ranged from 0.45 to 0.78. The strongest agreement was found for the sub-question that asked only those who slept with the light on whether they left it on all night. The poorest agreement was found for recall of whether participants had ever slept with an eye mask (0.45). However, only a small number of women reported this habit (*n* = 12). Good agreement was found for subjective sleep quality, duration of sleep on work and non-work days, diagnosis of sleep disorders, use of melatonin, and falling asleep with the light on. Moderate agreement was found for ever having experienced a period of sleep disturbance and number of such periods experienced.

Overall test-retest reliability of the sleep table was less than ideal. Although, on average, sleep loss per year and symptom-severity scores were the same at the 2 time points, the wide LOA(r) and the fair to moderate ICC indicate a need for improvement. In particular, the significant dependence found for symptom severity indicates a complex relationship between the difference and the mean of symptoms with increasing magnitude of symptom severity score. However, the β coefficient for dependence was relatively small (−0.19), and the LOA(r) analysis of skewed data will tend to give limits that are too far apart rather than too close, so we are not likely to have a falsely optimistic view of the agreement.^22^

Comparison with test-retest reliability of other sleep questionnaires is difficult. Although Devine et al identified 22 sleep questionnaires in their meta-analysis, only 8 were assessed for test-retest reliability. While none of those 8 questionnaires assessed recall of sleep data from more than 1 month prior, the Jenkins sleep problems scale^23^ and the Pittsburgh sleep quality index^12^ did contain questions or domains similar to those evaluated on the BCEES questionnaire. Both of these studies assessed test-retest reliability by using Pearson product-moment correlations, which are not directly comparable to the ICC and LOA(r) methods used in this study. Pearson correlations were not used in this study, as they have been shown to be inadequate for assessing test-retest reliability.^19^^,^^22^ The Pearson correlation indicates the strength of a relationship between 2 variables but not the agreement between them, and good correlation may be present even when agreement is poor.^22^ ICC and LOA(r) have been shown to be better tools for measuring the reliability of continuous variables.^19^ Unlike correlation, the LOA(r) method does not provide a single number representing agreement, but presents a number of statistics that must be interpreted in light of each other and the wider scope of the clinical consequences.

The present findings indicate that some changes to the questionnaire—specifically to the questions associated with the sleep table—are warranted. Of the questions on sleep habits, the small number of people who reported ever using a mask to cover their eyes while sleeping, and the poor reliability of the question, suggest that it could be modified to ask about usually using a mask, or even excluded from future versions of the questionnaire. The sleep table asked participants to identify and describe periods of sleep disturbance that lasted 2 weeks or longer. The questionnaire included examples of significant life events such as the birth of children, divorce, bereavement, and illness as prompts to help participants recall events in their life that might be associated with periods of sleep disturbance. While the 2-week minimum timeframe was chosen based on a conservative definition of insomnia,^24^ the 2-week time frame might have been too specific for accurate recall in this context. Future versions of the questionnaire should consider increasing the minimum period of disturbance when collecting information on lifetime history of sleep disturbance. Little is known about the duration of poor sleep that is likely to affect long-term health outcomes; thus, there is no established minimum threshold appropriate for identifying exposure to sleep disturbance. In delineating insomnia into acute and chronic variants, a minimum duration of 6 months of sleep disturbance has been used as a threshold for chronic insomnia,^24^ which may be a useful value for adjusting the BCEES questionnaire.

Another option for improving the reliability of the sleep table may be to incorporate fuzzy range estimates rather than point estimates.^25^ The format of standard sleep questionnaires requires respondents to provide a single value to represent their subjective estimate of sleep. Gehrman and colleagues suggest allowing respondents to provide a range estimate, so that participants can communicate information on their habits and the stability of their estimates. For example, in the sleep table, instead of asking participants to specify a single number for how many hours of sleep they were getting during the period of disturbance, a fuzzy response format would ask them to identify a range, eg, “During the period of disturbance I would get between __ and __ hours of sleep per night.” Gehrman et al suggest that the fuzzy response format may be better suited to the cognitive processes that underlie quantitative estimates of recalled sleep behavior. A test-retest study of fuzzy and point estimates for total sleep time and sleep latency found that fuzzy estimates were the same or better than point estimates.^25^ However, the potential advantages of fuzzy response formats are still speculative and would need to be weighed against the financial and psychological burden of the additional data collection.

The female study population was a limitation of this study. While survey studies of sleep quality have shown that women self-report higher rates of insomnia as compared with men,^24^ the reliability studies reviewed by Devine did not report their test-retest results by sex.^13^ Replicating the present study in men is important to improve the generalizability of the questionnaire. In addition, assessing the validity of the questionnaire is necessary.

In summary, this study found that the test-retest reliability of general sleep questions was good and that future epidemiologic studies of sleep could reliably expand the number of domains of sleep quality they assess to include duration of sleep on work and non-work days, subjective sleep quality, falling asleep with the light on, and experiencing periods of sleep disturbance. However, reliability decreased as participants were asked for increasing detail on specific periods of sleep disturbance, and changes to the sleep-table portion of the questionnaire are warranted.

## References

[1] Kronholm E, Partonen T, Laatikainen T, Peltonen M, Härmä M, Hublin C, Trends in self-reported sleep duration and insomnia-related symptoms in Finland from 1972 to 2005: a comparative review and re-analysis of Finnish population samples. J Sleep Res. 2008;17:54–62 10.1111/j.1365-2869.2008.00627.x 18275555

[2] Pires ML, Benedito-Silva AA, Mello MT, Del Giglio S, Pompeia C, Tufik S Sleep habits and complaints of adults in the city of Sao Paulo, Brazil, in 1987 and 1995. Braz J Med Biol Res. 2007;40:1505–15 10.1590/S0100-879X2006005000170 17934647

[3] Byles JE, Mishra GD, Harris MA, Nair K The problems of sleep for older women: changes in health outcomes. Age Ageing. 2003;32:154–63 10.1093/ageing/32.2.154 12615558

[4] Wingard DL, Berkman LF Mortality risk associated with sleeping patterns among adults. Sleep. 1983;6:102–7 6878979 10.1093/sleep/6.2.102

[5] Knutson KL, Spiegel K, Penev P, Van Cauter E The metabolic consequences of sleep deprivation. Sleep Med Rev. 2007;11:163–78 10.1016/j.smrv.2007.01.002 17442599 PMC1991337

[6] Kojima M, Wakai K, Kawamura T, Tamakoshi A, Aoki R, Lin Y, Sleep patterns and total mortality: a 12-year follow-up study in Japan. J Epidemiol. 2000;10:87–93 10.2188/jea.10.87 10778032

[7] Hublin C, Partinen M, Koskenvuo M, Kaprio J Sleep and mortality: a population-based 22-year follow-up study. Sleep. 2007;30:1245–53 17969458 10.1093/sleep/30.10.1245 PMC2266277

[8] Verkasalo PK, Lillberg K, Stevens RG, Hublin C, Partinen M, Koskenvuo M, Sleep duration and breast cancer: a prospective cohort study. Cancer Res. 2005;65:9595–600 10.1158/0008-5472.CAN-05-2138 16230426

[9] Kawakami N, Takatsuka N, Shimizu H Sleep disturbance and onset of type 2 diabetes. Diabetes Care. 2004;27:282–3 10.2337/diacare.27.1.282 14694011

[10] Nilsson PM, Rööst M, Engström G, Hedblad B, Berglund G Incidence of diabetes in middle-aged men is related to sleep disturbances. Diabetes Care. 2004;27:2464–9 10.2337/diacare.27.10.2464 15451917

[11] Akerstedt T, Hume K, Minors D, Waterhouse J The subjective meaning of good sleep, an intraindividual approach using the Karolinska Sleep Diary. Percept Mot Skills. 1994;79:287–96 10.2466/pms.1994.79.1.287 7991323

[12] Buysse DJ, Reynolds CF 3rd, Monk TH, Berman SR, Kupfer DJ The Pittsburgh Sleep Quality Index: a new instrument for psychiatric practice and research. Psychiatry Res. 1989;28:193–213 10.1016/0165-1781(89)90047-4 2748771

[13] Devine EB, Hakim Z, Green J A systematic review of patient-reported outcome instruments measuring sleep dysfunction in adults. Pharmacoeconomics. 2005;23:889–912 10.2165/00019053-200523090-00003 16153133

[14] Ikehara S, Iso H, Date C, Kikuchi S, Watanabe Y, Wada Y, Association of sleep duration with mortality from cardiovascular disease and other causes for Japanese men and Women: the JACC study. Sleep. 2009;32:295–301 19294949 10.1093/sleep/32.3.295 PMC2647783

[15] Kakizaki M, Inoue K, Kuriyama S, Sone T, Matsuda-Ohmori K, Nakaya N, Sleep duration and the risk of prostate cancer: the Ohsaki Cohort Study. Br J Cancer. 2008;99:176–8 10.1038/sj.bjc.6604425 18542076 PMC2453016

[16] McElroy JA, Newcomb PA, Titus-Ernstoff L, Trentham-Dietz A, Hampton JM, Egan KM Duration of sleep and breast cancer risk in a large population-based case-control study. J Sleep Res. 2006;15:241–9 10.1111/j.1365-2869.2006.00523.x 16911025

[17] Patel SR, Ayas NT, Malhotra MR, White DP, Schernhammer ES, Speizer FE, A prospective study of sleep duration and mortality risk in women. Sleep. 2004;27:440–4 15164896 10.1093/sleep/27.3.440

[18] Kripke DF, Garfinkel L, Wingard DL, Klauber MR, Marler MR Mortality associated with sleep duration and insomnia. Arch Gen Psychiatry. 2002;59:131–6 10.1001/archpsyc.59.2.131 11825133

[19] Watson PF, Petrie A Method agreement analysis: A review of correct methodology. Theriogenology. 2010;73:1167–79 10.1016/j.theriogenology.2010.01.003 20138353

[20] Erren TC Does light cause internal cancers? The problem and challenge of an ubiquitous exposure. Neuroendocrinol Lett. 2002;23 Suppl 2:55–64 12163851

[21] Bland JM, Altman DG Measuring agreement in method comparison studies. Stat Methods Med Res. 1999;8:135–60 10.1191/096228099673819272 10501650

[22] Bland JM, Altman DG Statistical methods for assessing agreement between two methods of clinical measurement. Lancet. 1986;1:307–10 10.1016/S0140-6736(86)90837-8 2868172

[23] Jenkins CD, Stanton BA, Niemcryk SJ, Rose RM A scale for the estimation of sleep problems in clinical research. J Clin Epidemiol. 1988;41:313–21 10.1016/0895-4356(88)90138-2 3351539

[24] Kryger M, Roth T, Dement WC, editors. Principles and practice of sleep medicine. 4th ed. Philadelphia: Elsevier Saunders; 2005.

[25] Gehrman P, Matt GE, Turingan M, Dinh Q, Ancoli-Israel S Towards an understanding of self-reports of sleep. J Sleep Res. 2002;11:229–36 10.1046/j.1365-2869.2002.00306.x 12220319

